# Assistive products and the Sustainable Development Goals (SDGs)

**DOI:** 10.1186/s12992-016-0220-6

**Published:** 2016-11-29

**Authors:** Emma Tebbutt, Rebecca Brodmann, Johan Borg, Malcolm MacLachlan, Chapal Khasnabis, Robert Horvath

**Affiliations:** 1 GATE Group, Essential Medicines & Health Products, World Health Organization, Geneva, Switzerland; 2 Social Medicine and Global Health, Lund University, Lund, Sweden; 3 Centre for Global Health, Trinity College Dublin, Dublin 2, Ireland; 4 Centre for Rehabilitation Studies, Stellenbosch University, Stellenbosch, South Africa; 5 Olomouc University Social Health Institute, Palacky University Olomouc, Olomouc, Czech Republic; 6 United States Agency for International Development, Washington D.C, USA

**Keywords:** Assistive products, Assistive technology, Sustainable Development Goals, SDGs, Limitations, People with disabilities, Global Cooperation on Assistive Technology, GATE

## Abstract

The Sustainable Development Goals (SDGs) have placed great emphasis on the need for much greater social inclusion, and on making deliberate efforts to reach marginalized groups. People with disabilities are often marginalized through their lack of access to a range of services and opportunities. Assistive products can help people overcome impairments and barriers enabling them to be active, participating and productive members of society. Assistive products are vital for people with disabilities, frailty and chronic illnesses; and for those with mental health problems, and gradual cognitive and physical decline characteristic of aging populations. This paper illustrates how the achievement of each of the 17 SDGs can be facilitated by the use of assistive products. Without promoting the availability of assistive products the SDGs cannot be achieved equitably. We highlight how assistive products can be considered as both a mediator and a moderator of SDG achievement. We also briefly describe how the Global Cooperation on Assistive Technology (GATE) is working to promote greater access to assistive products on a global scale.

## Background

The 2030 Agenda for Sustainable Development has a tremendous scope, spanning the three dimensions of economic, social and environmental development. Considered by the United Nations (UN) to be “a universal call to action to end poverty, protect the planet and ensure that all people enjoy peace and prosperity,” [1] its 17 Sustainable Development Goals (SDGs) will stimulate global action over the next 15 years.

The SDGs build on the Millennium Development Goals (MDGs) and focus on the key gaps in the progress made by the MDGs. At the heart of this is the pledge that “no-one will be left behind” and that governments will endeavour to reach “the furthest behind first” [2]. Persons with disabilities and older people, especially women, are among the groups of people who are most likely to remain left behind, despite collectively numbering between one and two billion [3, 4]. They are also the two largest groups of people who need assistive products. Without assistive products people are often excluded and locked into poverty and isolation; also increasing the impact of functional decline, disease and disability on the person, his/her family and on society.

Older people are living longer and have the potential to contribute to family and society in many ways. However, this potential for contribution is heavily dependent on good health. For many older people today added years are often lived with disability, mostly as a result of chronic disease [5].

Persons with disabilities have poorer health outcomes, lower education achievements and less economic participation than people without disabilities [3]. They are overrepresented among the poor, and are vulnerable in situations of natural and man-made disasters as well as in conflicts [6]. Children with disabilities experience poorer health, limited opportunities for education and economic opportunities and encounter greater inequalities than children without disabilities [7]; UNESCO has estimated that literacy rates of women and girls with disabilities are as low as 1% [8]. Persons with disabilities face disproportionate unemployment, depriving societies of an estimated 1.37 to 1.94 trillion US dollars in annual loss of GDP [9]. A study of ten low- and middle-income countries concluded that the losses constitute 3-7% of GDP [10].

Assistive products can greatly reduce inequalities experienced by all people living with impairments - children and adults with disabilities, and those living with chronic conditions and functional decline - by enabling them to be productive and participate in all areas of life. Achieving the SDGs and leaving no-one behind will not be possible if the people who need essential assistive products do not have access to them.

Assistive products, such as wheelchairs, artificial limbs, spectacles, hearing aids, pill organizers, and accessible information communication technology (ICT), have been developed to maintain or improve the functioning and independence of people with impairments [11]. Assistive products enable people to live healthy, productive, independent, and dignified lives, and to participate in education, the labour market and civic life. They are also important for primary and secondary prevention and management of noncommunicable and communicable diseases, like diabetes and leprosy, respectively. Moreover, they reduce the need for formal health and support services, long-term care and the work of caregivers [12]. The diversity of need for assistive products extends across the life span, across the continuum of health care and across all domains of human activity; representing a very large variety of products.

Access to good quality and affordable assistive products has been mandated by the Convention on the Rights of Persons with Disabilities (CRPD) for ten years but still only 10% of people in need of assistive products have access to them [12]. If this urgent need is not addressed, the percentage of people with access will decrease, as demand increases and access to services remains stagnant. The need is acute everywhere, especially in low-and middle-income countries. To support countries in their efforts to comply with the CRPD, the World Health Organization (WHO) launched the Global Cooperation on Assistive Technology (GATE) in 2014 in partnership with organizations of and for persons with disabilities; UN agencies, donor agencies, professional organizations, academia and industry. With the SDGs comes another international mandate and a platform to further raise awareness and build on existing efforts to improve access. The objective of this paper therefore is to highlight the importance of population-wide access to assistive products as a prerequisite for and facilitator of achieving the SDGs.

We used our own familiarity with the literature and where necessary undertook a literature search (using Google Scholar, Pubmed and Psychinfo) to identity relevant examples of assistive products being used in a way that was related to achievement of each of the SDGs. Table 1 lists the 17 SDGs and for each goal provides a conceptualisation for how assistive products are relevant. Each conceptualisation is illustrated with an example relating to one of the specific targets associated with each goal.

**Table 1:** The relevance of assistive products for achievement of the SDGs

*SDG 1. End poverty in all its forms everywhere*
*Conceptualization:* Poverty is both a significant cause and consequence of impairment and disability. Assistive products are powerful enablers for people with impairments to overcome poverty. *Target 1.2:* By 2030, reduce at least by half the proportion of men, women and children of all ages living in poverty in all its dimensions according to national definitions. *Example:* A study in Bangladesh found that the use of hearing aids and wheelchairs amongst people with hearing and ambulatory impairments respectively was predictive of reduced poverty (Borg et al., [15]).
*SDG 2. End hunger, achieve food security and improved nutrition and promote sustainable agriculture*
*Conceptualization:* Assistive products enable people with impairments to have the opportunity to contribute to the production of food. *Target 2.3:* Increase the agricultural productivity and incomes of small-scale food producers, in particular women and family farmers (among others, through non-farm employment). *Example:* Victims of landmines are often farmers from low and middle-income countries. When a farmer loses a limb in a landmine incident, a prosthesis enables him or her to continue to produce food. Families affected by landmines are 40% more likely to have difficulty obtaining adequate food (Walsh & Walsh [16]).
*SDG 3. Ensure healthy lives and promote well-being for all at all ages*
*Conceptualization:* Assistive products compensate for impairments, reduce the health and social consequences of gradual functional decline and are key for primary and secondary prevention of many health problems. *Target 3.4:* By 2030, reduce by one third premature mortality from non-communicable diseases through prevention and treatment and promote mental health and well-being. *Example:* Therapeutic footwear for diabetes reduces the incidence of foot ulcers, preventing lower limb amputations, and the associated implications and costs for the individual and for health systems (Bus et al., [17]).
*SDG 4. Ensure inclusive and equitable quality education and promote lifelong learning opportunities for all*
*Conceptualization:* Assistive products play a powerful role in both ensuring students with impairments access education and in supporting educational achievement. *Target 4a:* Build and upgrade education facilities that are child, disability and gender sensitive, and provide safe, non-violent, inclusive and effective learning environments for all. *Example:* Assistive products (including augmentative and alternative communication (AAC) devices, switches, touch screens and alternative keyboards) enable children with severe disabilities to communicate effectively with their teachers and peers, fostering learning and participation (Alquraini & Gut [18]).
*SDG 5. Achieve gender equality and empower all women and girls*
*Conceptualization:* Assistive products are essential for many women and girls with impairments to have gender equality, including equal rights, universal access to sexual and reproductive health, and for full participation. Additionally, assistive products can reduce the need for caregivers, roles which fall mostly to women and girls and consequently limit other opportunities for their full participation in society. *Target 5.b:* Enhance the use of enabling technology, in particular information and communications technology, to promote the empowerment of women *Example:* Women with disabilities in South Australia identified the importance of accessible ICT to reduce isolation, to access information, and to contribute to decision making within their communities (Jennings [19]).
*SDG 6. Ensure availability and sustainable management of water and sanitation for all*
*Conceptualization:* Assistive products enable people with impairments to access clean water and sanitation services. *Target 6.2:* By 2030, achieve access to adequate and equitable sanitation and hygiene for all and end open defecation, paying special attention to the needs of women and girls and those in vulnerable situations. *Example:* Grab rails, wheelchairs, ramps, and toilet chairs all enable access to toilets. A study in South Africa found that a lack of accessible toilets prevented university students who use wheelchairs from fully participating in lectures and university life (Losinsky et al. [20]).
*SDG 7. Ensure access to affordable, reliable, sustainable and modern energy for all*
*Conceptualization:* Assistive products enable people with impairments to have access to affordable and clean energy, be productive users of energy and enable them to pay for it, contributing economically. *Target 7.1*: By 2030, ensure universal access to affordable, reliable and modern energy services. *Example:* In the United Kingdom, unaffordable energy bills disproportionately affect older people. Changing energy provider can save one household £200 each year on energy bills but 60% of households age 65 and over have never switched (Age Action Alliance [21]). A leading British charity has developed written and auditory information to make it easier for older people to switch to the most affordable energy supplier, but for many of them this information will only be accessible with spectacles or hearing aids (Age UK: Love later life [22]).
*SDG 8. Promote sustained, inclusive and sustainable economic growth, full and productive employment and decent work for all*
*Conceptualization:* Assistive products enable people with impairments to have the opportunity to participate in the workforce, earn a living and contribute to the economy. *Target 8.5*: By 2030, achieve full and productive employment and decent work for all women and men, including for young people and persons with disabilities, and equal pay for work of equal value. *Example:* Assistive products (including adapted telephones, wheelchairs, magnifiers and adapted computer equipment) remove barriers to employment for workers with disabilities, resulting in substantial benefits to productively and self-esteem (Yeager et al., [23]).
*SDG 9. Build resilient infrastructure, promote inclusive and sustainable industrialization and foster innovation*
*Conceptualization:* Assistive products need to be an integral component of all infrastructure in order for infrastructure to be inclusive for all. *Target 9c:* Significantly increase access to information and communications technology and strive to provide universal and affordable access to the Internet in least developed countries by 2020. *Example:* Language software products can assist students with learning disabilities to learn how to read and write. Without access to the Internet, such products are not available, which hinders learning and restricts or prevents moving ahead with education (GAATES: Global Accessibility News [24]).
*SDG 10. Reduce inequality within and among countries*
*Conceptualization:* Assistive products greatly reduce inequalities by enabling people with impairments to participate in all areas of life. *Target 10.2:* By 2030, empower and promote the social, economic and political inclusion of all, irrespective of age, sex, disability, race, ethnicity, origin, religion or economic or other status *Example:* Underutilization of assistive products can delay successful transitions into independent living and community participation for adolescents and young adults with Spina Bifida (Johnson et al. [25]).
*SDG 11. Make cities and human settlements inclusive, safe, resilient and sustainable*
*Conceptualization:* Assistive products enable many people with impairments to access their cities and communities, including public transport systems. *Target 11.2:* By 2030, provide access to safe, affordable, accessible and sustainable transport systems for all, improving road safety, notably by expanding public transport, with special attention to the needs of those in vulnerable situations, women, children, persons with disabilities and older persons *Example:* People with impairments often describe lack of accessible transportation as a barrier to accessing services and social contact; barriers can be physical, cognitive, communication or environmental, among others, and can include issues such as lack of ramps, inaccessible timetable information and payment systems (Roberts & Babinard [26]).
*SDG 12. Ensure sustainable consumption and production patterns*
*Conceptualization:* Everyone, everywhere needs access to information for sustainable development and lifestyles. Assistive products enable people with impairments to access mainstream information channels. *Target 12.8*: Ensure that people everywhere have the relevant information and awareness for sustainable development and lifestyles. *Example:* Television is a primary source of information about sustainable lifestyles. Captioning displays enable viewers who are deaf and hard of hearing to have full access to programmes (National Institute on Deafness and Other Communication Disorders (NIDCD), [27]).
*SDG 13. Take urgent action to combat climate change and its impacts*
*Conceptualization:* Access to assistive products is important for strengthening resilience and adaptive capacity for people with impairments, especially in the event of natural disasters, a predictable outcome of global climate change. There is also an increased incidence of disease and injury as a result of extreme weather events, which leads to an increased need for assistive products (Rataj et al, [28]). *Target 13.1*: Strengthen resilience and adaptive capacity to climate-related hazards and natural disasters in all countries. *Example:* The Third UN World Conference on Disaster Risk Reduction was hailed by participants as the first international meeting of its kind to provide a wide range of accessibility features, in order to ensure that people with disabilities are consulted on plans and strategies for managing disaster risk. Closed captioning, sign language interpretation, wheelchair accessible venues and transport, accessible documents and Braille displays enabled more than 200 people with impairments to actively participate as delegates, speakers, panellists, and contributors (UNISDR, [29]).
*SDG 14. Conserve and sustainably use the oceans, seas and marine resources for sustainable development*
*Conceptualization:* Just as on land, assistive products enable people with impairments the opportunity to contribute to the use of marine resources, and to benefit from the tourism and self-development potential of such environments. *Target 14.7:* By 2030, increase the economic benefits to Small Island developing States and least developed countries from the sustainable use of marine resources, including through sustainable management of fisheries, aquaculture and tourism. *Example:* Presbyopia (blurred near vision) affects most people beyond middle age and can be simply corrected with spectacles, enabling people to continue to contribute to family life and livelihoods (Holden, et al., [30]). A core activity of small-scale fishing industries is the maintenance and repair of fishing nets – an ideal task for older family members if they have access to spectacles.
*SDG 15. Protect, restore and promote sustainable use of terrestrial ecosystems, sustainably manage forests, combat desertification, and halt and reverse land degradation and halt biodiversity loss*
*Conceptualization:* Assistive products enable people with impairments to contribute to protecting, restoring and promoting sustainable use of the environment. *Target 15.3*: By 2030, combat desertification, restore degraded land and soil, including land affected by desertification, drought and floods, and strive to achieve a land degradation-neutral world. *Example:* With access to wheelchairs, a group of people with disabilities in Malawi were able to start a sustainable farming business, growing mushrooms in a greenhouse, which required no fertilizers or chemicals and which did not add to the degradation of the land locally. All parts of the process can be done from a wheelchair, no hard labour or digging is needed and mushrooms are light and easy to transport. (World Health Organization, [31]).
*SDG 16. Promote peaceful and inclusive societies for sustainable development, provide access to justice for all and build effective, accountable and inclusive institutions at all levels*
*Conceptualization:* In order for societies to be inclusive, all people who need assistive products need to be able to access them. *Target 16.3*: Promote the rule of law at the national and international levels and ensure equal access to justice for all *Example:* Documents in Braille enable a person who is blind to access information in order to have equitable access to justice (UN Partnership on the Rights of Persons with Disability, [32]).
*SDG 17. Strengthen the means of implementation and revitalize the global partnership for sustainable development*
*Conceptualization:* As illustrated by the examples above, assistive products are important facilitators of sustainable development. Strong global partnerships are crucial for ensuring that essential assistive products are available and affordable, and that everyone, everywhere can access them. *Target 17.16*: Enhance the global partnership for sustainable development, complemented by multi-stakeholder partnerships that mobilize and share knowledge, expertise, technology and financial resources, to support the achievement of the sustainable development goals in all countries, in particular developing countries. *Example:* In October 2015, China hosted an Asia-Europe High-Level Meeting on Disability and Global Conference on Assistive Devices and Technology (Ministry of Foreign Affairs of the People’s Republic of China, [33]). The German Chancellor and Chinese Premier initiated a collaboration towards developing the manufacturing capacity of high-quality affordable assistive products – a prerequisite for the universal access needed to meet the SDGs.

**Fig. 1:**
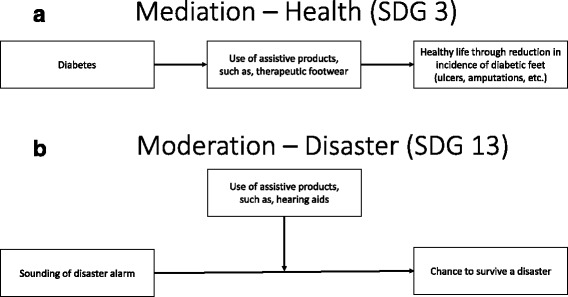
**a** Mediation – Health (SDG 3). **b** Moderation – Disaster (SDG 13)

Table 1 highlights how assistive products can help to achieve the SDGs. Some of these may be more intuitively clear than others. For instance, assistive products may *mediate* the relationship between an intervention to achieve a particular SDG and actual outcomes associated with it. Figure 1a illustrates this mediating relationship for SDG 3, which focuses on ensuring healthy lives and promoting well-being. People who have diabetes, and who have a need for and are provided with therapeutic footwear (an assistive product), can often avoid ulcers and in some cases the need for amputation; thus allowing them the potential to experience better quality of life and wellbeing. So in this example the achievement of the goal works directly through – is mediated by – provision of an appropriate assistive product.

At other times an intervention to achieve an SDG may be effective in its own right, but unavailable to all who may benefit from it. For instance, in regard to SDG 13 and its reference to disaster mitigation; Fig. 1b illustrates how a community may install an alarm system to warn people of the need to evacuate in the case of an impending flood. Without hearing aids many members of the community will not be able to hear and respond to the warning. This is a case of assistive products *moderating* the effectiveness of an intervention. With appropriate provision of assistive products the intervention is more effective; with inadequate provision of assistive products it is less effective. Thus, at a population level, assistive products can facilitate - or *moderate* - interventions that are not necessarily concerned with impairment, but for which the provision of assistive products can strengthen their effect. The moderating and mediating effects of assistive products are relevant across all areas of life – at both individual and population levels.

This paper illustrates how achievement of the SDGs and the use of assistive products can be conceptualised; and provides examples of how achieving specific targets can be facilitated through the use of assistive products. Thus, universal access to assistive products represents a fundamental criterion and missing link between people who are likely to remain left behind and achievement of the goals. They are a necessity not only for the achievement of each SDG, but they also facilitate the relationship between the goals. For example, many people with impairments need assistive products in order to access education and jobs and therefore escape poverty and hunger.

Attempting to achieve the SDGs without appropriate population-level access to assistive products would not only be inherently discriminatory, but would also negate the fundamental principle of equity underscored in each goal. Indeed very few interventions have the potential to generate such significant cross-cutting impact, or the potential multiplier effect across different areas of life, for instance, from health and education to employment and justice.

In an effort to address the huge unmet need for assistive products, in May 2016, GATE launched the first WHO Priority Assistive Products List (APL) [13] with an aim of increasing access to high quality and affordable assistive products. The APL includes 50 priority assistive products, selected through an extensive consultative process which included users and potential users of assistive products as well as experts and other stakeholders.

The APL must be complemented by the establishment of appropriate national infrastructure. In addition to a national Priority Assistive Products List, three additional components are necessary: policy, service provision and trained personnel. GATE is developing tools to address these components. A policy framework will provide guidance for countries to develop assistive products policy and programmes. It will address existing barriers in relation to financing, procurement, standards, training of personnel and service provision. A model of service provision will give guidance on the integration of assistive products service provision into existing health or other services. Finally, a community-level training package will support the development of workforce capacity. Trained personnel are essential for the proper prescription, fitting, maintenance, user training and follow up of assistive products [12].

## Conclusion

Assistive products need to be prioritized by development partners and governments as an essential component for inclusive sustainable development. Universal access to essential assistive products will not only create advances in human rights, but will also benefit society economically, socially and environmentally. As the number of people in need of assistive products globally increases, so does the urgency with which Member States must address this long neglected situation. To ensure no-one is really left behind and that the SDGs are achieved equitably, universal access to high-quality affordable assistive products needs to be prioritized, both across sectors and in the planning and implementation of rehabilitative and assistive services [14].
